# Morbidity and factors associated with frailty in post-COVID-19 elderly patients attended at a reference center

**DOI:** 10.1590/0034-7167-2023-0454

**Published:** 2025-01-10

**Authors:** Ely Carlos Pereira de Jesus, Victor Guilherme Pereira, Zilá Aparecida Soares Pereira, Maria Suzana Marques, Cristiane Vieira da Silva, Leila das Graças Siqueira, Luciana Colares Maia, Antônio Prates Caldeira

**Affiliations:** I Universidade Estadual de Montes Claros. Montes Claros, Minas Gerais, Brazil

**Keywords:** Health Services for the Aged, Epidemiology, Frailty, COVID-19, Secondary Care, Servicios de Salud para Ancianos, Epidemiología, Fragilidad, COVID-19, Atención Secundaria de Salud

## Abstract

**Objective:**

To assess the morbidity profile and identify factors associated with frailty syndrome in post-COVID-19 elderly patients treated at the only Reference Center for Elderly Health Care in northern Minas Gerais.

**Methods:**

This is a case series study, utilizing the Clinical-Functional Vulnerability Index-20 (CFVI-20) and Comprehensive Geriatric Assessment (CGA) to characterize and evaluate the health condition of the group. To define the variables associated with frailty, a multivariate analysis was conducted.

**Results:**

The study included 204 elderly individuals, with a predominance of females (63.7%). The variables associated with frailty were cognitive impairment (OR: 2.95; 95% CI: 1.12-7.80; p=0.029), the presence of five or more comorbidities (OR: 11.55; 95% CI: 2.22-60.01; p=0.004), and impairment in instrumental activities of daily living (OR: 41.97; 95% CI: 5.47-321.93; p<0.001).

**Conclusions:**

The results of this study highlight the need for a well-established and prepared coordination of integrated care to meet the demands of the post-COVID-19 elderly population.

## RESUMEN

**Objetivo:**

Evaluar el perfil de morbidez e identificar los factores asociados con el síndrome de fragilidad en ancianos post-COVID-19 atendidos en el único Centro de Referencia en Asistencia a la Salud del Anciano en el norte de Minas Gerais.

**Métodos:**

Estudio de serie de casos, utilizando el Índice de Vulnerabilidad Clínico-Funcional-20 (IVCF-20) y la Evaluación Geriátrica Ampliada (AGA) para la caracterización y evaluación de la condición de salud del grupo. Para definir las variables asociadas a la fragilidad, se realizó un análisis multivariado.

**Resultados:**

Participaron en el estudio 204 ancianos, con predomínio del sexo femenino (63,7%). Las variables asociadas a la fragilidad fueron: deterioro cognitivo (OR: 2,95; IC95%: 1,12-7,80; p=0,029), presencia de cinco o más morbilidades (OR: 11,55; IC95%: 2,22-60,01; p=0,004) y deterioro en las actividades instrumentales de la vida diaria (OR: 41,97; IC95%: 5,47-321,93; p<0,001).

**Conclusiones:**

Los resultados de este estudio resaltan la necesidad de una coordinación del cuidado integrado, bien establecida y preparada para atender las demandas de la población anciana post-COVID-19.

## INTRODUCTION

In Brazil, as in many other regions around the world, the elderly population has proven to be the most vulnerable to COVID-19, suffering from high mortality rates due to the disease^(1, 2, 3)^. Research has found that among the survivors of this group, there is a significant incidence of sequelae and complications that still require further study^(2, 3, 4)^. Additionally, Frailty Syndrome (FS) — a condition frequently observed among the elderly^(5)^ — is an independent predictor of an increased risk of death associated with COVID-19^(6, 7)^.

It is worth noting that, even before the COVID-19 pandemic, FS was already a significant global public health issue among the elderly^(8)^. The process of epidemiological transition has led to the replacement of infectious diseases with chronic non-communicable diseases, shifting the burden of morbidity and mortality from younger groups to the elderly population, particularly those with clinical-functional frailty^(9)^.

Although there is no universally established definition for this condition, the literature describes frailty as a syndrome with multiple causes and risk factors, characterized by a decrease in physical strength and endurance, as well as impairment of physiological functions, which increases susceptibility to negative outcomes such as cognitive decline, decompensation and complications of comorbidities, institutionalization, hospitalization, physical-functional dependency, and/or death^(10, 11)^.

It is understood that the COVID-19 pandemic coincided with the significant phenomenon of population aging, which is considered a major demographic event of the 21st century, both nationally and globally. This phenomenon has negatively impacted healthcare systems, overburdening and in some cases collapsing them, as they are primarily utilized by the elderly population^(3, 12, 13, 14)^. An international study revealed that frailty was associated with a higher risk of developing severe forms of COVID-19, post-infection sequelae, and/or exacerbation and decompensation of chronic diseases among elderly patients^(7)^.

In this context, other studies around the world that have investigated the association between frailty and the increased risk of developing critical forms of COVID-19 highlight that the assessment of clinical-functional status aids in the timely identification of elderly individuals with a high risk of severe disease^(7, 15)^, with a high potential for complications, loss of functionality, hospitalization, and death^(3, 15, 16)^. Therefore, it is highly likely that COVID-19 is a triggering factor for frailty and the exacerbation/decompensation of comorbidities among the elderly, given the vulnerabilities inherent to the aging process^(7, 8, 17)^.

It is evident that the national literature is still evolving on this important topic. Therefore, evaluating the relationships between FS and COVID-19 is, in this context, a necessity and a responsibility for healthcare services, managers, and health professionals. This observation is particularly true given the needs of public health systems and the functional sequelae associated with the socioeconomic vulnerabilities of users, which underlines the importance of providing rehabilitation for the elderly during and after infection with SARS-CoV-2 (Severe Acute Respiratory Syndrome Coronavirus 2)^(15, 16)^.

## OBJECTIVE

To evaluate the morbidity profile and identify factors associated with frailty syndrome in post-COVID-19 elderly patients treated at the only Reference Center for Elderly Health Care in northern Minas Gerais.

## METHODS

### Ethical Aspects

The study was conducted in accordance with the guidelines established by Resolution 466/12 of the National Health Council (CNS). The research project was approved by the Research Ethics Committee (CEP) of the State University of Montes Claros, and data collection was carried out with the agreement of the specialized center through the Institutional Consent Form (TCI).

### Study Design, Period, and Location

This is a case series study with exploratory and analytical evaluation, conducted with a group of elderly individuals who were treated at the Reference Center for Elderly Health Care (CRASI) after a confirmed diagnosis of COVID-19, following the criteria of the Strengthening the Reporting of Observational Studies in Epidemiology (STROBE) checklist^(18)^.

The Reference Center for Elderly Health Care (CRASI) Eny Faria de Oliveira is responsible for the referred care of 86 municipalities in the northern macro-region of the state of Minas Gerais, Brazil, with a total estimated population of 1,676,413 inhabitants^(19)^. It is noteworthy that, considering only the municipality of Montes Claros (MG) — the most significant urban center in the region and where the service is located — the specialized center serves as a reference for 132 Family Health Strategy (ESF) units.

The service is supported by a multidisciplinary care team composed of geriatricians, nurses, physiotherapists, speech therapists, dentists, nutritionists, physical educators, and nursing technicians. It includes a support house, performs specialized examinations, offers individualized therapeutic plans, and provides counter-referral to family health teams (eSF), in addition to conducting continuous education activities and providing technical-pedagogical support (matrix support). In this way, it integrates with the Primary Health Care (PHC) service, the Health Care Network (RAS), and hospital care, aiming to connect the different levels of care.

### Population or sample, inclusion and exclusion criteria

The population of this study consisted of elderly individuals of both sexes who were referred by Primary Health Care (PHC) professionals after a confirmed diagnosis of COVID-19 through testing (rapid antigen test, RT-PCR, or serological test), regardless of the severity of their condition or level of frailty, for follow-up, continued care, and rehabilitation in secondary care services.

The sampling process was one of convenience, though not intentional, based on the sequential identification and selection of elderly individuals affected by COVID-19 who were treated following the reopening of services that had previously been interrupted due to the pandemic. No exclusion criteria were applied. It should be noted that no statistical calculation was performed, as the intent was to evaluate the entire group of elderly individuals referred to the reference center.

### Study Protocol

Data collection was conducted using the MAIS VIDA system, a software tool utilized by healthcare professionals to record care provided at CRASI. The elderly patients treated at the specialized center were administered a socioeconomic and demographic questionnaire, a health-related factors identification questionnaire, the Clinical-Functional Vulnerability Index (IVCF-20)^(20)^, the Mini-Mental State Examination (MMSE)^(21)^, the Katz Index of Independence in Activities of Daily Living^(22)^, and the Pfeffer Functional Activities Questionnaire^(23)^. Information was collected between October 2021 and August 2022.

The socioeconomic and demographic characteristics considered for evaluation included: sex, age, education level, marital status, self-reported skin color, employment status, individual income, number of children, and household arrangement. The following variables were also assessed: Body Mass Index (BMI), with reference values defined as underweight when ≤ 22.0; normal weight when between 22.1 and 26.9; and overweight/obesity when ≥ 27.0^(24)^; presence of comorbidities; and, according to self-report, the number of medications in use, vaccination status, and the need for hospitalization due to COVID-19.

The IVCF-20 is a screening questionnaire for identifying potentially frail elderly individuals, developed and validated in Brazil in 2016, and is used to identify individuals at risk of clinical-functional frailty. The stratification of frailty status is defined by the following scores: robust - 0 to 6 points; pre-frail - 7 to 14 points; and frail - ≥ 15 points^(20)^.

The MMSE is employed to detect cognitive decline. The scores indicating cognitive impairment are determined based on education levels, as follows: for illiterate individuals, < 13 points indicate cognitive impairment; for those with 1 to 4 years of education, < 18 points indicate cognitive impairment; and for those with five or more years of education, < 26 points indicate cognitive impairment^(21)^.

The Katz Index is an instrument used to evaluate the geriatric process, with the objective of assessing functional status based on the Basic Activities of Daily Living (BADL), related to the patient’s self-care and essential needs. It scores from zero to six, with zero representing dependency for all activities and six representing independence for all functions (bathing, dressing, eating, toileting, transferring, and maintaining continence)^(22)^.

Additionally, the Pfeffer Functional Activities Questionnaire is used to assess the integrity of Instrumental Activities of Daily Living (IADLs). It evaluates the elderly individual’s ability to perform more complex activities and indicates the level of independence within the community. Scores ≥ 6 points indicate impairments in IADLs^(23)^.

### Analysis of results and statistics

For data analysis, descriptive statistics were initially synthesized to characterize the absolute and relative frequencies of the events studied. Pearson’s chi-square test or Fisher’s exact test was performed to compare characteristics between male and female elderly individuals, and additionally to analyze the association or independence of the estimates investigated, adopting a significance level of 5% (p<0.05).

To evaluate factors associated with frailty syndrome, bivariate analyses were conducted, and variables that were associated up to the 20% level (p≤0.20) were further analyzed jointly using binary logistic regression. In the final model, only variables associated at the 5% level were retained, with the Odds Ratios and their respective 95% confidence intervals (95% CI) reported. All data were processed using the Statistical Package for the Social Sciences (SPSS) software, version 28.0.

## RESULTS

The study included 204 elderly individuals, of whom 130 (63.7%) were women and 74 (36.3%) were men. The majority were aged between 60 and 79 years (77.5%). In terms of education, 133 (64.7%) of the elderly reported having up to four years of schooling.

Statistically significant differences were observed between male and female participants regarding marital status, number of children, and number of cohabitants. These and other characteristics of the evaluated group are presented in Table 1.

**Table 1:** Socioeconomic and demographic characterization of post-COVID-19 elderly individuals by sex, treated at a reference center, Montes Claros, Minas Gerais, Brazil, 2021

Socioeconomic and demographic situation	Male (n=74)		Female (n=130)		Total (N=204)		*p* value
	n	%	n	%	n	%	
Age (years)							0.913
60 - 79	57	77.0	101	77.7	158	77.5	
≥ 80	17	23.0	29	22.3	46	22.5	
Education (in years of schooling)							0.291
Illiterate	11	11.9	28	21.5	39	19.1	
1 to 4 years of schooling	53	71.6	79	60.8	132	64.7	
≥ 5 years of schooling	10	13.5	23	17.7	33	16.2	
Marital status							0.021
With a partner	51	68.9	68	52.3	119	58.3	
Without a partner	23	31.1	62	47.7	85	41.7	
Skin color (self-reported)							0.893
Black	6	8.1	12	9.2	18	8.8	
Brown	53	71.6	89	68.5	142	69.6	
White/Other	15	20.3	29	22.3	44	21.6	
Employment status							0.255
Regular employment	6	8.1	4	3.1	10	4.9	
Unemployed	6	8.1	9	3.9	15	7.4	
Retired	62	83.8	117	90.0	179	87.4	
Elderly income (in minimum wages)							0.541
< 1 minimum wage	8	34.8	15	65.2	23	11.3	
≥ 1 minimum wage	66	36.5	115	63.5	181	87.7	
Number of children							0.014
None	8	10.8	4	3.1	12	5.9	
1 to 5 children	45	60.8	79	60.8	124	60.8	
≥ 6 children	21	29.1	47	36.1	68	35.1	
Household arrangement							0.002
Lives alone	6	8.1	18	13.8	24	11.8	
1 to 3 people	64	86.5	83	62.8	147	72.1	
≥ 4 people	4	5.4	29	22.3	33	16.2	

The health profile of the investigated group reveals that more than 70% of the elderly individuals have limitations or impairments in at least one basic activity of daily living (BADL); nearly half of the group has impairments in instrumental activities of daily living (IADL); a similar percentage also has a high BMI. Almost 80% of the group reported having three or more comorbidities, with just over a quarter of the group reporting five or more comorbidities.

The results show significant differences between men and women regarding functionality, with a higher proportion of women having some functional impairment (p=0.015). Additionally, it is noted that women had a higher frequency of polypharmacy (p=0.046). Furthermore, more than half of the elderly had an incomplete vaccination schedule against SARS-CoV-2, and about 20% had been hospitalized due to COVID-19, but without significant differences between men and women (Table 2).

**Table 2:** Clinical and functional characterization of post-COVID-19 elderly individuals by sex, treated at a reference center, Montes Claros, Minas Gerais, Brazil, 2021

Socioeconomic and demographic situation	Male (n=74)		Female (n=130)		Total (N=204)		*p* value
	n	%	n	%	n	%	
Mini-Mental State Examination (MMSE)							0.195
Not impaired	58	78.4	91	70.0	149	73.0	
Impaired	16	21.6	39	30.0	55	27.0	
Impaired BADL*							0.015
4 to 6	6	8.1	7	5.4	13	6.4	
1 to 3	40	54.1	96	73.8	136	66.7	
None	28	37.8	27	20.8	55	26.9	
IADL**							
Not impaired	39	52.7	68	52.3	107	52.4	0.957
Impaired	35	47.3	62	47.7	97	47.6	
Body Mass Index (BMI)							0.056
Underweight	12	16.2	11	8.5	23	11.3	
Normal weight/Eutrophy	35	47.3	51	39.2	86	42.2	
Overweight/obesity	27	36.5	68	52.3	95	46.5	
Number of comorbidities							0.054
≤ 2	23	31.1	22	16.9	45	22.1	
3 to 4	36	48.6	71	54.6	107	52.4	
≥ 5	15	20.3	37	28.5	52	25.5	
Medications in use							0.046
0 ato 4	52	70.3	70	53.8	122	59.8	
≥ 5	22	29.7	60	46.2	82	40.2	
COVID-19 vaccination status							0.354
Incomplete	36	48.6	72	55.4	108	53.0	
Complete	38	51.4	58	44.6	96	47.0	
Hospitalization due to COVID-19							0.963
Yes	15	20.3	26	20.0	41	20.1	
No	59	79.7	104	80.0	163	79.9	

Notes: *BADL - Basic Activities of Daily Living (Katz Index); **IADL - Instrumental Activities of Daily Living (Pfeffer Scale).

The assessment of the group regarding frailty status revealed that 93 elderly individuals (45.6%) were classified as robust, 80 (39.2%) were considered pre-frail, and 31 (15.2%) were classified as frail. Table 3 shows the association between the characteristics of the elderly individuals treated at the specialized center and their frailty status, based on a bivariate analysis.

**Table 3:** Factors associated with frailty among post-COVID-19 elderly individuals treated at a reference center, Montes Claros, Minas Gerais, Brazil, 2021 (bivariate analysis)

Variables	IVCF-20 Assessment			*p* value
	Frailty n (%)	Non-Frailty n (%)	Total (N=204)	
Sex				0.614
Male	10 (13.5)	64 (86.5)	74 (36.3%)	
Female	21 (16.2)	109 (83.8)	130 (63.7%)	
Age (years)				0.061
60 - 79	11 (23.9)	35 (76.1)	46 (22.5%)	
≥ 80	20 (12.7)	138 (87.3)	158 (77.5%)	
Education (in years of schooling)				0.790
Illiterate	7 (17.9)	32 (82.1)	39 (19.1%)	
1 to 4 years of schooling	20 (15.2)	112 (84.8)	132 (64.7%)	
≥ 5 years of schooling	4 (12.1)	29 (87.9)	33 (16.2%)	
Marital status				0.106
With a partner	17 (20.0)	68 (80.0)	85 (41.7%)	
Without a partner	14 (11.8)	105 (88.2)	119 (58.3%)	
Skin color (self-reported)				0.745
Black/Brown	25 (15.6)	135 (84.4)	160 (78.4%)	
White/Other	6 (13.6)	38 (86.4)	44 (21.6%)	
Elderly income (in minimum wages)				0.031
< 1	7 (30.4)	16 (69.6)	23 (11.3%)	
≥ 1	24 (13.3)	157 (86.7)	181 (88.7%)	
Impaired BADL*				<0.001
≥ 2	29 (33.0)	59 (67.0)	88 (43.1%)	
< 2	2 (1.9)	114 (98.3)	116 (56.9%)	
Body Mass Index (BMI)				0.574
Overweight/obesity (≥ 27)	13 (13.7)	82 (86.3)	95 (45.6%)	
Eutrophy/Underweight (< 27)	18 (16.5)	91 (83.5)	109 (53.4%)	
IADL**				<0.001
Impaired	30 (31.3)	66 (68.7)	96 (47.1%)	
Not impaired	1 (0.9)	107 (99.1)	108 (52.9%)	
Mini-Mental State Examination (MMSE)				0.001
Impaired	16 (29.1)	39 (70.9)	55 (27.0%)	
Not Impaired	15 (10.1)	134 (89.9)	149 (73.0%)	
Number of Comorbidities				<0.001
≥ 5	18 (34.6)	34 (65.4)	52 (25.4%)	
3 to 4	11 (10.3)	96 (89.7)	107 (52.5%)	
≤ 2	2 (4.4)	43 (95.6)	45 (22.1%)	
Medications in use				0.010
≥ 5	25 (20.5)	97 (79.5)	122 (59.8%)	
< 5	6 (7.3)	79 (92.7)	82 (40.2%)	
COVID-19 vaccination status				0.073
Incomplete	21 (19.4)	87 (80.6)	108 (52.9%)	
Complete	10 (10.4)	86 (89.6)	96 (47.1%)	
Hospitalization due to COVID-19				0.067
Yes	10 (24.4)	31 (75.6)	41 (20.1%)	
No	21 (12.9)	142 (87.1)	163 (79.9%)	

Notes: *BADL - Basic Activities of Daily Living (Katz Index); **IADL - Instrumental Activities of Daily Living (Pfeffer Scale).

Variables that were associated up to the 20% level (p<0.20) were evaluated together using binary logistic regression. The variables that remained in the final model (Table 4), assuming a significance level of 5%, were: impaired score on the mental examination (OR: 2.95; 95% CI: 1.12-7.80; p=0.029); presence of five or more comorbidities (OR: 11.55; 95% CI: 2.22-60.01; p=0.004); and impaired score in performing instrumental activities of daily living (OR: 41.97; 95% CI: 5.47-321.93; p<0.001).

**Table 4:** Variables associated with frailty syndrome among post-COVID-19 elderly individuals treated at a reference center, Montes Claros, Minas Gerais, Brazil, 2021 (multiple analysis by binary logistic regression)

Variables	Frailty (IVCF-20 ≥ 15)	
	OR* (95% CI**)	*p* value
Mini-Mental State Examination (MMSE)		0.029
Not impaired	2.95 (1.12-7.80)	
Impaired	1.00	
Number of comorbidities		0.004
≥ 5	11.55 (2.22-60.01)	
3 to 4	3.51 (1.30-9.49)	
≤ 2	1.00	
Instrumental Activities of Daily Living (IADL)		<0.001
Impaired	41.97 (5.47-321.93)	
Not impaired	1.00	

Notes: *Odds Ratio; **95% Confidence Interval.

## DISCUSSION

This study identifies the main socioeconomic, demographic, and health characteristics of post-COVID-19 elderly individuals treated at the only Reference Center for Elderly Health Care (CRASI) in the northern macro-region of the state of Minas Gerais, Brazil. The group evaluated is relatively homogeneous in terms of socioeconomic and demographic characteristics; however, statistically significant differences were observed between male and female elderly individuals regarding marital status, number of children, and household arrangement.

The health profile of the elderly reveals a significant percentage of dependence on functional capacity and autonomy, considering the impairment of basic and instrumental activities of daily living. The presence of multimorbidities and the frequent use of five or more medications (polypharmacy) also highlights the need for special care for post-COVID-19 elderly individuals due to their inherent clinical vulnerability^(3, 4)^.

Due to the nature of the study, it was not possible to determine whether the reported morbidities began before or after the confirmed SARS-CoV-2 infection. Nonetheless, it is worth noting that a meta-analysis of longitudinal studies found that COVID-19 can precipitate the onset of certain morbid conditions^(6)^.

A higher percentage of women without partners, with more children, and living alone was observed compared to elderly men. Similarly, other studies addressing the aging process have also found that elderly women without partners are more common than elderly men^(24, 25)^.

Regarding health conditions, the results also show differences between the sexes in terms of functionality, with a higher proportion of women experiencing some level of functional impairment, a finding that is also recorded in other investigations^(24, 26)^.

Additionally, polypharmacy, another relevant factor associated with elderly health, was also found to be more prevalent among women, with higher rates of five or more medications used by women. This is consistent with the literature, as a longitudinal study conducted in 70 Brazilian municipalities found significant disparities between elderly men and women, with considerably higher proportions of polypharmacy among women^(27)^.

Other studies conducted in Europe, the United States, and Brazil warn that polypharmacy is a common condition among the elderly and is often associated with multiple chronic conditions. Furthermore, a significant prevalence of negative outcomes due to this condition has been observed, including higher rates of mortality, falls, hospitalizations, adverse drug reactions, and increased healthcare costs^(28, 29, 30)^.

The multivariate model revealed that cognitive impairment, the presence of five or more comorbidities, and impairment in IADLs were statistically associated with frailty status. The association between frailty and cognitive impairment is consistently reported in the literature, underscoring the need for early detection of cognitive changes to understand their dynamic evolution and their impact on the clinical-functional status of the elderly^(31, 32, 33)^.

In this regard, a prospective cohort study conducted with elderly individuals at an academic medical center in São Paulo observed a higher proportion of frail elderly among those with cognitive impairment, with this group being larger than that observed among elderly individuals without this condition. Additionally, the authors warn that frail patients with cognitive impairment have a higher risk of mortality (Hazard Ratio (HR): 4.38; 95% CI: 1.95-9.87)^(33)^.

Cognitive decline is frequently observed with the progression of the aging process and may be driven by genetic factors, socioeconomic factors, lifestyle habits, and the presence of comorbidities. In this context, frailty has been internationally recognized as a relevant condition. A longitudinal cohort study conducted in Asia found that elderly individuals with cognitive decline had a 3.77 times greater probability (Odds Ratio) of developing clinical-functional frailty (95% CI: 1.42-9.99)^(34)^.

It is noteworthy that both conditions share the same pathophysiological mechanisms of inflammatory activation and neuroendocrine dysregulation, which consequently contribute to the coexistence of these two conditions^(34)^. Therefore, elderly individuals with cognitive impairment are considered at risk for frailty^(31, 32, 33, 34, 35)^.

Similar to this study, a cross-sectional investigation conducted with elderly patients at a specialized gerontology center in the interior of São Paulo found a correlation between frailty and a higher number of comorbidities (r=0.539; p=0.004)^(31)^. The presence of multimorbidities is linked to greater restrictions in daily living activities. Although it does not necessarily indicate the frailty of an individual, with advancing age, this factor can accelerate the development of frailty and indicate a higher risk for the onset of this syndrome^(36)^.

Another variable that also showed a statistically significant association with frailty was the impairment of IADLs. A cross-sectional study conducted with elderly individuals in São Paulo reinforced this association, concluding that more frail elderly individuals exhibit worse functional conditions compared to less frail ones (r=−0.57; p=0.002)^(37)^. These findings suggest that, in general, as the transition to a frailty state progresses, elderly individuals display poorer performance in both basic and instrumental activities of daily living^(38)^.

It is noteworthy that a significant portion of the elderly individuals investigated in the present study fall into the pre-frail category. These individuals are considered to be in the process of becoming frail, which is concerning given the lack of a clear definition of the condition’s evolutionary nature. It is essential that healthcare professionals promote increased vigilance and integrated care for this group, as a longitudinal cohort study conducted at a geriatric specialty outpatient center in Minas Gerais followed elderly patients for twelve months and found that 33.3% of non-frail elderly individuals with cognitive impairment transitioned to frailty^(32)^.

Focusing specifically on the context of COVID-19, it is important to note that most of the elderly individuals in this study had an incomplete vaccination status, and a significant portion of the evaluated group required hospitalization due to the disease. It was not possible to directly assess the impact of the disease on frailty status, considering that the patients were not evaluated beforehand.

Initially, a higher proportion of frail elderly individuals might have been expected in the study, given that other research points to a close relationship between frailty and COVID-19^(6, 7, 15, 16)^. However, it is important to consider that more frail elderly individuals experienced severe outcomes, with prolonged hospitalizations and post-COVID-19 mortality^(3, 7, 16)^. The process of selecting the elderly individuals involved in this study is a limiting factor for more specific considerations about the relationship between COVID-19 and frailty, as it was conducted based on specific and voluntary demand.

In the context of the COVID-19 pandemic, international investigations have warned that clinical-functional frailty is correlated with a higher risk of developing severe forms of the disease, postinfection sequelae, and/or exacerbation and decompensation of chronic diseases among elderly patients, particularly when associated with hospitalization^(1, 3, 7)^. Other inquiries also highlight that the hospitalization of frail elderly individuals affected by COVID-19, with multimorbidities, cognitive decline, and functional impairment, significantly reduces survival rates^(3, 7, 33, 39, 40)^.

### Study Limitations

The results of this study should be considered in light of some limitations. As previously mentioned, this is a case series with specific analyses, which do not allow for the determination of causality. Additionally, memory and selection biases should be considered, given that the research data were obtained through the service’s patient care records, which were documented based on the accounts of the elderly individuals treated at the specialized center for clinical evaluation after COVID-19 infection. Since the evaluations took place after the disease had manifested, they only refer to elderly survivors who were able to attend the service for care, limiting the generalizability of the findings.

### Contributions to Nursing, Health, or Public Policy

Despite these limitations, the observed results underscore the urgent need for comprehensive care for the elderly, focusing on the specificities of frailty syndrome and the post-COVID-19 condition. Additionally, they highlight the need for ongoing education and technical-pedagogical support (matrix support) for the services and healthcare professionals involved in this context of care.

## CONCLUSIONS

The health profile of the group is characterized primarily by elderly individuals who have limitations in functionality and autonomy, along with a high percentage of associated comorbidities. Predominantly, the evaluated group was found to be in a frail/pre-frail condition.

The variables associated with frailty in elderly individuals post-SARS-CoV-2 infection were cognitive impairment, the presence of five or more comorbidities, and impairment in instrumental activities of daily living.

Therefore, it becomes relevant to evaluate the post-COVID-19 condition as a potential additional marker of frailty, especially among the elderly. These results highlight the need for well-established and integrated care coordination to meet the demands of the post-COVID-19 elderly population.

## References

[1] Barbosa IR, Galvão MHR, Souza TA, Gomes SV, Medeiros AA, Lima KC (2020). Incidence of and mortality from COVID-19 in the older Brazilian population and its relationship with contextual indicators: an ecological study.. Rev Bras Geriatr Gerontol..

[2] Romero DE, Myzy J, Damacena GN, Souza NA, Almeida WS, Szwarcwald CL (2021). Idosos no contexto da pandemia da COVID-19 no Brasil: efeitos nas condições de saúde, renda e trabalho.. Cad Saúde Pública..

[3] Bonanad C, García-Blas S, Tarazona-Santabalbina F, Sanchis J, Bertomeu-González V, Fácila L (2020). The effect of age on mortality in patients with COVID-19: a meta-analysis with 611,583 subjects.. J Am Med Dir Assoc..

[4] Huang C, Wang Y, Li X, Ren L, Zhao J, Hu Y (2020). Clinical features of patients infected with 2019 novel coronavirus in Wuhan, China.. Lancet..

[5] Carneiro JA, Cardoso RR, Durães MS, Guedes MCA, Santos FL, Costa FM (2017). Frailty in the elderly: prevalence and associated factors.. Rev Bras Enferm..

[6] Feng Z, Lugtenberg M, Franse C, Fang X, Hu S, Jin C (2017). Risk factors and protective factors associated with incident or increase of frailty among community-dwelling older adults: a systematic review of longitudinal studies.. PLoS One..

[7] Zhang XM, Jiao J, Cao J, Huo XP, Zhu C, Wu XJ (2021). Frailty as a predictor of mortality among patients with COVID-19: a systematic review and meta-analysis.. BMC Geriatr..

[8] Buckinx F, Rolland Y, Reginster JY, Ricour C, Petermans J, Bruyère O (2015). Burden of frailty in the elderly population: perspectives for a public health challenge.. Arch Public Health..

[9] Verma M, Kalra S (2020). Epidemiological transition in South-East Asia and its Public Health Implications.. J Pak Med Assoc [Internet].

[10] Hoogendijk EO, Afilalo J, Ensrud KE, Kowal P, Onder G, Fried LP (2019). Frailty: implications for clinical practice and public health.. Lancet..

[11] Tavares JPA, Sá-Couto PMF, Pedreira LC (2022). Predictors of frailty in older people users of Primary Health Care.. Rev Bras Enferm..

[12] Yenilmez MI (2015). Economic and social consequences of population aging the dilemmas and opportunities in the twenty-first century.. Appl Res Qual Life..

[13] Veras RP, Oliveira M (2018). Envelhecer no Brasil: a construção de um modelo de cuidado.. Ciênc saúde Coletiva..

[14] Campos MR, Schramm JMA, Emmerick ICM, Rodrigues JM, Avelar FG, Pimentel TG (2020). Carga de doença da COVID-19 e de suas complicações agudas e crônicas: reflexões sobre a mensuração (DALY) e perspectivas no Sistema Único de Saúde.. Cad Saúde Pública..

[15] Hussien H, Nastasa A, Apetrii M, Nistor I, Petrovic M, Covic A (2021). Different aspects of frailty and COVID-19: points to consider in the current pandemic and future ones.. BMC Geriatr..

[16] Nalbandian A, Sehgal K, Gupta A, Madhavan MV, McGroder C, Stevens JS (2021). Post-acute COVID-19 syndrome.. Nat Med..

[17] Gómez-Belda AB, Fernández-Garcés M, Mateo-Sanchis E, Madrazo M, Carmona M, Piles-Roger L (2021). COVID-19 in older adults: what are the differences with younger patients?. Geriatr Gerontol Int..

[18] Von Elm E, Altman DG, Egger M, Pocock SJ, Gøtzsche PC, Vandenbroucke JP, STROBE Initiative (2008). The Strengthening the Reporting of Observational Studies in Epidemiology (STROBE) statement: guidelines for reporting observational studies.. J Clin Epidemiol..

[19] Secretaria de Estado de Saúde de Minas Gerais (2019). Deliberação CIB-SUS/MG nº 3.013, de 23 de outubro de 2019. Adscrição dos municípios de minas gerais por microrregião e macrorregião de saúde, conforme o ajuste de 2019 do plano diretor de regionalização SUS/MG.

[20] Moraes EN, Carmo JA, Moraes FL, Azevedo RS, Machado CJ, Montilla DER (2016). Clinical-Functional Vulnerability Index-20 (IVCF-20): rapid recognition of frail older adults.. Rev Saúde Pública..

[21] Folstein MF, Folstein SE, McHugh PR (1975). “Mini-mental state”: a practical method for grading the cognitive state of patients for the clinician.. J Psychiatr Res..

[22] Katz S, Ford AB, Moskowitz RW, Jackson BA, Jaffe MW (1963). Studies of illness in the aged, the index of ADL: a standardized measure of biological ans psychosocial function.. JAMA..

[23] Pfeffer RI, Kurosaki TT, Harrah CH Jr, Chance JM, Filos S (1982). Measurement of functional activities in older adults in the community.. J Gerontol..

[24] Williams JS, Norström F, Ng N (2017). Disability and ageing in China and India: decomposing the effects of gender and residence, results from the WHO study on global AGEing and adult health (SAGE).. BMC Geriatr..

[25] Melo NCV, Teixeira KMD, Barbosa TL, Montoya AJA, Silveira BM (2016). Arranjo domiciliar de idosos no Brasil: análises a partir da Pesquisa Nacional por Amostra de Domicílios (2009).. Rev Bras Geriatr Gerontol..

[26] Anand A, Syamala TS, Sk MK, Bhatt N (2020). Understanding frailty, functional health and disability among older persons in india: a decomposition analysis of gender and place of resident.. J Res Health Sci..

[27] Seixas BV, Freitas GR (2021). Polypharmacy among older Brazilians: prevalence, factors associated, and sociodemographic disparities (ELSI-Brazil).. Pharm Pract (Granada)..

[28] Xue L, Boudreau RM, Donohue JM, Zgibor JC, Marcum ZA, Costacou T (2021). Persistent polypharmacy and fall injury risk: the Health, Aging and Body Composition Study.. BMC Geriatr..

[29] Midão L, Giardini A, Menditto E, Kardas P, Costa E (2018). Polypharmacy prevalence among older adults based on the survey of health, ageing and retirement in Europe.. Arch Gerontol Geriatr..

[30] Sousa CR, Coutinho JFV, Freire Neto JB, Barbosa RGB, Marques MB, Diniz JL (2022). Factors associated with vulnerability and fragility in the elderly: a cross-sectional study.. Rev Bras Enferm..

[31] Barbosa GC, Caparrol AJS, Melo BRS, Medeiros TJ, Ottaviani AC, Gratão ACM (2022). Fatores correlacionados à fragilidade de idosos em atenção ambulatorial: diferença entre grupos etários.. Esc Anna Nery..

[32] Alencar MA, Oliveira AC, Figueiredo LC, Dias JMD, Dias RC (2018). Prevalence and transition to frailty in older adults with cognitive impairment: a 1-year cohort study.. Geriatr Gerontol Aging..

[33] Aprahamian I, Suemoto CK, Aliberti MJR, Fortes Fo SQ, Melo JA, Lin SM (2018). Frailty and cognitive status evaluation can better predict mortality in older adults?. Arch Gerontol Geriatr..

[34] Jacobs JM, Cohen A, Ein-Mor E, Maaravi Y, Stessman J (2011). Frailty, cognitive impairment and mortality among the oldest old.. J Nutr Health Aging..

[35] Grden CRB, Rodrigues CRB, Cabral LPA, Reche PM, Bordin D, Borges PKO (2019). Prevalence and factors associated with the frailty in elderly patients attended to anoutpatient care specialty clinics.. Rev Eletron Enferm..

[36] Closs VE, Ziegelmann PK, Gomes I, Schwanke CHA (2016). Frailty and geriatric syndromes in elderly assisted in primary health care.. Acta Scienti Health Sci..

[37] Cerqueira Leite J, Machado de Jesus IT, Souza Orlandi F, Silvana Zazzetta M (2019). Fragilidade, cognição, depressão e funcionalidade de idosos em condomínio habitacional.. Estud Interdiscip Envelhec..

[38] Lustosa LP, Marra TA, Pessanha FPAS, Freitas JC, Guedes RC (2013). Fragilidade e funcionalidade entre idosos frequentadores de grupos de convivência em Belo Horizonte, MG.. Rev Bras Geriatr Gerontol..

[39] Nunes BP, Souza ASS, Nogueira J, Andrade FB, Thumé E, Teixeira DSC (2020). Multimorbidade e população em risco para COVID-19 grave no Estudo Longitudinal da Saúde dos Idosos Brasileiros.. Cad Saúde Pública..

[40] Hubbard RE, Maier AB, Hilmer SN, Naganathan V, Etherton-Beer C, Rockwood K (2020). Frailty in the face of COVID-19.. Age Ageing..

