# Supplementary material for: Morbidity and factors associated with frailty in post-COVID-19 elderly patients attended at a reference center
Source: Rev Bras Enferm. 2025 Jan 10;77(Suppl 1):e20230454. doi: 10.1590/0034-7167-2023-0454 (PMC11726821; doi:10.1590/0034-7167-2023-0454)
Supplement: Translation

# Translation: Morbidade e fatores associados à fragilidade em idosos pós-COVID-19 atendidos em um centro de referência

**DOI:** 10.1590/0034-7167-2023-0454pt

**Authors:** Ely Carlos Pereira de Jesus, Victor Guilherme Pereira, Zilá Aparecida Soares Pereira, Maria Suzana Marques, Cristiane Vieira da Silva, Leila das Graças Siqueira, Luciana Colares Maia, Antônio Prates Caldeira

**Affiliations:** I Universidade Estadual de Montes Claros. Montes Claros, Minas Gerais, Brasil

**Keywords:** Serviços de Saúde para Idosos, Epidemiologia, Fragilidade, COVID-19, Atenção Secundária à Saúde

## Abstract

**Objetivo:**

Avaliar o perfil de morbidade e identificar os fatores associados à síndrome da fragilidade em idosos pós-COVID-19, atendidos no único Centro de Referência em Assistência à Saúde do Idoso no norte de Minas Gerais.

**Métodos:**

Estudo de série de casos, utilizando o Índice de Vulnerabilidade Clínico-Funcional-20 (IVCF-20) e a Avaliação Geriátrica Ampla (AGA) para caracterização e avaliação da condição de saúde do grupo. Para definição das variáveis associadas à fragilidade, conduziu-se análise multivariada.

**Resultados:**

Participaram do estudo 204 idosos, com predominância do sexo feminino (63,7%). As variáveis associadas à fragilidade foram: comprometimento cognitivo (OR: 2,95; IC95%: 1,12-7,80; p=0,029), presença de cinco ou mais morbidades (OR: 11,55; IC95%: 2,22-60,01; p=0,004) e comprometimento nas atividades instrumentais de vida diária (OR: 41,97; IC95%: 5,47-321,93; p<0,001).

**Conclusões:**

Os resultados deste estudo ressaltam a necessidade de uma coordenação do cuidado integrado, bem estabelecida e preparada para atender as demandas da população idosa pós-COVID-19.

## INTRODUÇÃO

No Brasil, assim como em diversas regiões do mundo, a população idosa demonstrou ser a mais vulnerável à COVID-19, sendo vítima de expressivos índices de mortalidade pela doença^(1, 2, 3)^. Investigações constataram que, entre os sobreviventes desse grupo, há um considerável registro de sequelas e complicações que ainda demandam novos estudos^(2, 3, 4)^. Adicionalmente, a Síndrome de Fragilidade (SF) — condição frequentemente registrada entre as pessoas idosas^(5)^ — representa um preditor independente para maior risco de óbito associado à COVID-19^(6, 7)^.

Vale ressaltar que, entre os idosos, a SF já representava, mesmo antes da pandemia de COVID-19, um importante problema de saúde pública global^(8)^. Destaca-se que o processo de transição epidemiológica repercute na substituição das doenças infectocontagiosas por morbidades crônicas não transmissíveis, o que gerou uma transição da carga de morbimortalidade dos grupos mais jovens para a população idosa, principalmente aqueles com fragilidade clínico-funcional^(9)^.

Embora não exista uma definição bem estabelecida para essa condição, a literatura descreve a fragilidade como uma síndrome de múltiplas causas e fatores de risco, caracterizada pela diminuição da força física e da resistência, além de gerar comprometimento de funções fisiológicas que provocam maior suscetibilidade para o desenvolvimento de desfechos negativos, como declínio cognitivo, descompensação e complicações de comorbidades, institucionalização, hospitalização, dependência físico-funcional e/ou óbito^(10, 11)^.

Compreende-se que a pandemia de COVID-19 coincidiu com a evolução do significativo fenômeno do envelhecimento populacional, apontado como um expressivo evento demográfico do século XXI, tanto em nível nacional quanto mundial, o qual reverbera negativamente, sobrecarregando e colapsando os sistemas de saúde, utilizados, em sua maioria, pela população idosa^(3, 12, 13, 14)^. Um estudo internacional revelou que a fragilidade esteve associada a um maior risco de desenvolver formas graves de COVID-19, sequelas pós-infecção e/ou agudização e descompensação de doenças crônicas entre pacientes idosos^(7)^.

Nessa direção, outras produções ao redor do mundo que investigaram a associação entre fragilidade e risco aumentado de desenvolver formas críticas de COVID-19 alertam que a avaliação da condição clínico-funcional auxilia no reconhecimento oportuno de idosos com elevada chance de doença grave^(7, 15)^, com alto potencial de complicações, perda da funcionalidade, hospitalização e óbito^(3, 15, 16)^. Assim, é muito provável que a COVID-19 seja um fator desencadeador da fragilidade e agudização/descompensação de morbidades entre pessoas idosas, considerando as vulnerabilidades inerentes ao processo de envelhecimento^(7, 8, 17)^.

Depreende-se que a literatura nacional ainda está em evolução sobre essa importante temática. Portanto, avaliar as relações entre a SF e a COVID-19 é, nesse contexto, uma necessidade e um compromisso dos serviços assistenciais, dos gestores e dos profissionais de saúde. Essa constatação é particularmente verdadeira diante das necessidades dos sistemas públicos de saúde e das sequelas funcionais correlacionadas às vulnerabilidades socioeconômicas dos usuários, o que fomenta recomendações relevantes para proporcionar a reabilitação do idoso durante e após a infecção pelo SARS-CoV-2 (*Severe Acute Respiratory Syndrome Coronavirus* 2)^(15, 16)^.

## OBJETIVO

Avaliar o perfil de morbidade e identificar os fatores associados à síndrome da fragilidade em idosos pós-COVID-19, atendidos no único Centro de Referência em Assistência à Saúde do Idoso no norte de Minas Gerais.

## MÉTODOS

### Aspectos éticos

O estudo foi conduzido em conformidade com as normativas estabelecidas pela Resolução 466/12 do Conselho Nacional de Saúde (CNS). O projeto de pesquisa foi aprovado pelo Comitê de Ética em Pesquisa (CEP) da Universidade Estadual de Montes Claros (Unimontes), e a coleta de dados ocorreu mediante a concordância do centro especializado por meio do Termo de Consentimento Institucional (TCI).

### Desenho, período e local do estudo

Trata-se de um estudo de série de casos, com avaliação exploratória e analítica, conduzido a partir de um grupo de pessoas idosas atendidas, após diagnóstico confirmado de COVID-19, em um Centro de Referência em Assistência à Saúde do Idoso (CRASI), seguindo os critérios da lista de verificação do *Strengthening the Reporting of Observational Studies in Epidemiology* (STROBE)^(18)^.

O Centro de Referência em Assistência à Saúde do Idoso (CRASI) Eny Faria de Oliveira é responsável pelo atendimento referenciado de 86 municípios da macrorregião norte do estado de Minas Gerais, Brasil, cuja população total é estimada em 1.676.413 habitantes^(19)^. Destaca-se que, considerando apenas o município de Montes Claros (MG) — núcleo urbano mais expressivo da região e onde se situa o serviço — o centro especializado é referência para 132 unidades de Estratégia Saúde da Família (ESF).

O serviço conta com uma equipe assistencial multiprofissional, composta por geriatras, enfermeiros, fisioterapeutas, fonoaudiólogos, odontólogos, nutricionistas, educadores físicos e técnicos de enfermagem. Possui uma casa de apoio, realiza exames especializados, oferece planos terapêuticos individualizados e contrarreferência às equipes de saúde da família (eSF), além de atividades de educação permanente e apoio técnico-pedagógico (matriciamento). Dessa forma, integra-se ao serviço de Atenção Primária à Saúde (APS), à Rede de Atenção à Saúde (RAS) e à assistência hospitalar, na tentativa de conectar os diferentes níveis de cuidado.

### População ou amostra, critérios de inclusão e exclusão

A população deste estudo foi composta por idosos, de ambos os sexos, encaminhados por profissionais da APS, após quadro confirmado de COVID-19 por exame (teste rápido de antígeno, RT-PCR ou sorológico), independentemente do nível de gravidade e de fragilidade, para acompanhamento, cuidado continuado e reabilitação no serviço de atenção secundária.

O processo de amostragem foi por conveniência, mas não intencional, fundamentado na identificação e seleção sequencial das pessoas idosas acometidas pela COVID-19 e atendidas a partir da reabertura dos serviços, previamente interrompidos em função da pandemia. Não foram aplicados critérios de exclusão. Ressalta-se que não foi realizado cálculo estatístico, considerando o interesse em avaliar todo o contingente de idosos encaminhados ao centro de referência.

### Protocolo do estudo

A coleta de dados ocorreu por meio de consulta ao sistema MAIS VIDA, *software* utilizado pelos profissionais de saúde para o registro de atendimentos no CRASI. Os idosos atendidos no centro especializado foram submetidos a um questionário socioeconômico e demográfico, a um questionário de identificação de fatores relacionados à saúde, ao Índice de Vulnerabilidade Clínico-Funcional (IVCF-20)^(20)^, ao Mini Exame do Estado Mental (MEEM)^(21)^, à escala de Katz^(22)^ e ao questionário de Pfeffer^(23)^. A coleta de informações foi realizada entre outubro de 2021 e agosto de 2022.

As características socioeconômicas e demográficas consideradas para avaliação foram: sexo, idade, escolaridade, estado conjugal, cor da pele (autorreferida), atividade laborativa, renda individual, número de filhos e arranjo domiciliar. Também foram avaliadas as seguintes variáveis: Índice de Massa Corporal (IMC), assumindo-se como valores de referência: baixo peso, quando ≤ 22,0; peso adequado, quando entre 22,1 e 26,9; e sobrepeso/obesidade, quando ≥ 27,0^(24)^; presença de morbidades; e, segundo autorrelato, número de medicamentos em uso, situação vacinal e necessidade de hospitalização por COVID-19.

O IVCF-20 é um questionário para triagem inicial de idosos potencialmente frágeis, desenvolvido e validado no Brasil em 2016, aplicado para identificar o indivíduo em fragilização clínico-funcional. A estratificação do estado de fragilidade é definida a partir da pontuação: robusto - 0 a 6 pontos; pré-frágil - 7 a 14 pontos; e frágil - ≥ 15 pontos^(20)^.

O MEEM é empregado para detecção de declínio cognitivo. Para definição dos escores que determinam o comprometimento cognitivo, foram observados os níveis de escolaridade, a saber: para analfabetos, < 13 pontos indicam cognição comprometida; para aqueles com 1 a 4 anos de estudo, < 18 pontos indicam cognição comprometida; e para aqueles com cinco ou mais anos de estudo, < 26 pontos indicam cognição comprometida^(21)^.

A escala de Katz é um instrumento para avaliação do processo geriátrico, com o objetivo de pesquisar o estado funcional a partir das Atividades Básicas da Vida Diária (ABVD), relacionadas ao autocuidado e às necessidades humanas do paciente. Apresenta escores de zero a seis, sendo que zero representa a condição de dependência para todas as atividades e seis a condição de independência para todas as funções (banhar-se, vestir-se, alimentar-se, ir ao banheiro, transferir-se, ter continência)^(22)^.

De modo complementar, o questionário de Pfeffer é utilizado para verificar a integridade das Atividades Instrumentais de Vida Diária (AIVD). Avalia a capacidade do idoso em desempenhar atividades mais complexas e demonstra o quanto o sujeito estudado é independente dentro da comunidade onde vive. Escores ≥ 6 pontos indicam comprometimentos nas AIVDs^(23)^.

### Análise dos resultados e estatística

Para análise dos dados, inicialmente, foram sintetizadas as estatísticas descritivas, para caracterização das frequências (absolutas e relativas) dos eventos estudados. Realizou-se o teste qui-quadrado de Person ou teste exato de Fisher para comparar as características entre idosos do sexo masculino e feminino e, adicionalmente, para a análise de associação ou independência das estimativas investigadas, adotando-se o nível de significância de 5% (p<0,05).

Para avaliação dos fatores associados à síndrome da fragilidade, foram desenvolvidas análises bivariadas e as variáveis que se mostraram associadas até o nível de 20% (p≤0,20), foram analisadas de forma conjunta por meio da regressão logística binária. No modelo final, foram mantidas apenas as variáveis associadas até o nível de 5%, registrando-se as *Odds Ratios* e os respectivos intervalos de confiança de 95% (IC95%). Todos os dados foram processados por meio do *software Statistical Package for the Social Sciences* (SPSS) versão 28.0.

## RESULTADOS

Participaram do estudo 204 idosos, sendo 130 (63,7%) mulheres e 74 (36,3%) homens. A maioria apresentava idade entre 60 a 79 anos (77,5%). Quanto à escolaridade, 133 (64,7%) idosos relataram até quatro anos de estudo.

Foram registradas diferenças estatisticamente significativas entre os sexos masculino e feminino em relação ao estado conjugal, número de filhos e número de coabitantes. Essas e outras características do grupo avaliado são exibidas na Tabela 1.

**Tabela 1:** Caracterização socioeconómica e demográfica de pessoas idosas pós-COVID-19, segundo sexo, atendidas em um centro de referência, Montes Claros, Minas Gerias, Brasil, 2021

Situação socioeconómica e demográfica	Masculino (n=74)		Feminino (n=130)		Total (N=204)		Valor de *p*
	n	%	n	%	n	%	
Idade (anos)							0,913
60 - 79	57	77,0	101	77,7	158	77,5	
≥ 80	17	23,0	29	22,3	46	22,5	
Escolaridade (em anos de estudo)							0,291
Analfabeto	11	11,9	28	21,5	39	19,1	
1 a 4 anos de estudo	53	71,6	79	60,8	132	64,7	
≥ 5 anos de estudo	10	13,5	23	17,7	33	16,2	
Estado conjugal							0,021
Com companheiro(a)	51	68,9	68	52,3	119	58,3	
Sem companheiro(a)	23	31,1	62	47,7	85	41,7	
Cor da pele (autorreferida)							0,893
Preta	6	8,1	12	9,2	18	8,8	
Parda	53	71,6	89	68,5	142	69,6	
Branca/outras	15	20,3	29	22,3	44	21,6	
Atividade Laborativa							0,255
Trabalho regular	6	8,1	4	3,1	10	4,9	
Desempregado	6	8,1	9	3,9	15	7,4	
Aposentado	62	83,8	117	90,0	179	87,4	
Renda do idoso (em salários-mínimos)							0,541
< 1 salário-mínimo	8	34,8	15	65,2	23	11,3	
≥ 1 salário-mínimo	66	36,5	115	63,5	181	87,7	
Número de filhos							0,014
Nenhum	8	10,8	4	3,1	12	5,9	
1 a 5 filhos	45	60,8	79	60,8	124	60,8	
≥ 6 filhos	21	29,1	47	36,1	68	35,1	
Arranjo domiciliar							0,002
Reside sozinho	6	8,1	18	13,8	24	11,8	
1 a 3 pessoas	64	86,5	83	62,8	147	72,1	
≥ 4 pessoas	4	5,4	29	22,3	33	16,2	

O perfil de saúde do grupo investigado revela que mais de 70% das pessoas idosas possuem limitação ou comprometimento de pelo menos uma atividade básica de vida diária (ABVD); quase metade do grupo possui comprometimento de atividades instrumentais de vida diária (AIVD); percentual similar registra, ainda, elevado IMC; quase 80% do contingente referiu possuir três ou mais morbidades, sendo que pouco mais de um quarto do grupo registrou cinco ou mais morbidades.

Os resultados registram distinções relevantes entre homens e mulheres em relação à funcionalidade, com maior proporção de mulheres com algum comprometimento de funcionalidade (p=0,015). Além disso, nota-se que o sexo feminino apresentou maior frequência de polifarmácia (p=0,046). Destaca-se, ainda, que mais da metade dos idosos apresentavam esquema vacinal incompleto contra o SARS-CoV-2 e cerca de 20% registravam internação por COVID-19, mas sem diferenças importantes entre os sexos masculino e feminino (Tabela 2).

**Tabela 2:** Caracterização clínica e funcional de pessoas idosas pós-COVID-19, segundo sexo, atendidas em um centro de referência, Montes Claros, Minas Gerais, Brasil, 2021

Fatores relacionados à saúde	Masculino (n=74)		Feminino (n=130)		Total (N=204)		Valor de *p*
	n	%	n	%	n	%	
Mini Exame do Estado Mental (MEEM)							0,195
Não comprometido	58	78,4	91	70,0	149	73,0	
Comprometido	16	21,6	39	30,0	55	27,0	
ABVD comprometidas*							0,015
4 a 6	6	8,1	7	5,4	13	6,4	
1 a 3	40	54,1	96	73,8	136	66,7	
Nenhuma	28	37,8	27	20,8	55	26,9	
AIVD**							
Não comprometidas	39	52,7	68	52,3	107	52,4	0,957
Comprometidas	35	47,3	62	47,7	97	47,6	
Índice de Massa Corporal (IMC)							0,056
Baixo peso	12	16,2	11	8,5	23	11,3	
Peso adequado/eutrofia	35	47,3	51	39,2	86	42,2	
Sobrepeso/obesidade	27	36,5	68	52,3	95	46,5	
Número de morbidades							0,054
≤ 2	23	31,1	22	16,9	45	22,1	
3 a 4	36	48,6	71	54,6	107	52,4	
≥ 5	15	20,3	37	28,5	52	25,5	
Medicamentos em uso							0,046
0 a 4	52	70,3	70	53,8	122	59,8	
≥ 5	22	29,7	60	46,2	82	40,2	
Situação vacinal contra a COVID-19							0,354
Incompleta	36	48,6	72	55,4	108	53,0	
Completa	38	51,4	58	44,6	96	47,0	
Hospitalização por COVID-19							0,963
Sim	15	20,3	26	20,0	41	20,1	
Não	59	79,7	104	80,0	163	79,9	

Notas: *ABVD - Atividades Básicas de Vida Diária (Escala de Katz); **AIVD - Atividades Instrumentais de Vida Diária (Escala de Pfeffer).

A avaliação do grupo em relação ao estado de fragilidade revelou que 93 idosos (45,6%) foram classificados como robustos, 80 (39,2%) foram considerados pré-frágeis e 31 (15,2%) foram classificados como frágeis. Tabela 3 demonstra a associação entre as características dos idosos assistidos no centro especializado e o estado de fragilidade, a partir de uma análise bivariada.

**Tabela 3:** Fatores associados à fragilidade entre idosos pós-COVID-19, atendidos em um centro de referência, Montes Claros, Minas Gerais, Brasil, 2021 (análise bivariada)

Variáveis	Avaliação IVCF-20			Valor de *p*
	Frágil n (%)	Não-Frágil n (%)	Total (N=204)	
Sexo				0,614
Masculino	10 (13,5)	64 (86,5)	74 (36,3%)	
Feminino	21 (16,2)	109 (83,8)	130 (63,7%)	
Idade (anos)				0,061
≥ 80	11 (23,9)	35 (76,1)	46 (22,5%)	
60 – 79	20 (12,7)	138 (87,3)	158 (77,5%)	
Escolaridade (em anos de estudo)				0,790
Analfabeto	7 (17,9)	32 (82,1)	39 (19,1%)	
1 a 4	20 (15,2)	112 (84,8)	132 (64,7%)	
≥ 5	4 (12,1)	29 (87,9)	33 (16,2%)	
Estado conjugal				0,106
Sem companheiro(a)	17 (20,0)	68 (80,0)	85 (41,7%)	
Com companheiro(a)	14 (11,8)	105 (88,2)	119 (58,3%)	
Cor da pele (autorreferida)				0,745
Preta/parda	25 (15,6)	135 (84,4)	160 (78,4%)	
Branca/outras	6 (13,6)	38 (86,4)	44 (21,6%)	
Renda do idoso (em salários-mínimos)				0,031
< 1	7 (30,4)	16 (69,6)	23 (11,3%)	
≥ 1	24 (13,3)	157 (86,7)	181 (88,7%)	
ABVD* comprometidas				<0,001
≥ 2	29 (33,0)	59 (67,0)	88 (43,1%)	
< 2	2 (1,9)	114 (98,3)	116 (56,9%)	
Índice de Massa Corporal (IMC)				0,574
Sobrepeso/obesidade (≥ 27)	13 (13,7)	82 (86,3)	95 (45,6%)	
Eutrofia/baixo peso (< 27)	18 (16,5)	91 (83,5)	109 (53,4%)	
AIVD**				<0,001
Comprometidas	30 (31,3)	66 (68,7)	96 (47,1%)	
Não comprometidas	1 (0,9)	107 (99,1)	108 (52,9%)	
Mini Exame do Estado Mental (MEEM)				0,001
Comprometido	16 (29,1)	39 (70,9)	55 (27,0%)	
Não Comprometido	15 (10,1)	134 (89,9)	149 (73,0%)	
Número de Morbidades				<0,001
≥ 5	18 (34,6)	34 (65,4)	52 (25,4%)	
3 a 4	11 (10,3)	96 (89,7)	107 (52,5%)	
≤ 2	2 (4,4)	43 (95,6)	45 (22,1%)	
Medicamentos em uso				0,010
≥ 5	25 (20,5)	97 (79,5)	122 (59,8%)	
< 5	6 (7,3)	79 (92,7)	82 (40,2%)	
Situação vacinal contra a COVID-19				0,073
Incompleta	21 (19,4)	87 (80,6)	108 (52,9%)	
Completa	10 (10,4)	86 (89,6)	96 (47,1%)	
Hospitalização por COVID-19				0,067
Sim	10 (24,4)	31 (75,6)	41 (20,1%)	
Não	21 (12,9)	142 (87,1)	163 (79,9%)	

Notas: *ABVD - Atividades Básicas de Vida Diária (Escala de Katz); **AIVD - Atividades Instrumentais de Vida Diária (Escala de Pfeffer).

As variáveis que se mostraram associadas até o nível de 20% (p<0,20) foram avaliadas, em conjunto, por meio da regressão logística binária. As variáveis que permaneceram no modelo final (Tabela 4), assumindo-se um nível de significância de 5%, foram: escore comprometido na avaliação do exame mental (OR: 2,95; IC95%: 1,12-7,80; p=0,029); presença de cinco ou mais morbidades (OR: 11,55; IC95%: 2,22-60,01; p=0,004); e escore comprometido para a realização de atividades instrumentais de vida diária (OR: 41,97; IC95%: 5,47-321,93; p<0,001).

**Tabela 4:** Variáveis associadas à síndrome da fragilidade entre idosos pós-COVID-19, atendidos em um centro de referência, Montes Claros, Minas Gerais, Brasil, 2021 (análise múltipla por regressão logística binária)

Variáveis	Fragilidade (IVCF-20 ≥ 15)	
	OR* (IC95%)	Valor de *p*
Mini Exame do Estado Mental (MEEM)		0,029
Comprometido	2,95 (1,12-7,80)	
Não Comprometido	1,00	
Número de morbidades		0,004
≥ 5	11,55 (2,22-60,01)	
3 a 4	3,51 (1,30-9,49)	
≤ 2	1,00	
Atividades Instrumentais de Vida Diária (AIVD)		<0,001
Comprometida	41,97 (5,47-321,93)	
Não comprometida	1,00	

Notas: *Odds Ratio; **Intervalo de Confiança de 95%

## DISCUSSÃO

Este estudo permite determinar as principais características socioeconômicas, demográficas e de saúde de idosos pós-COVID-19, atendidos no único Centro de Referência em Assistência à Saúde do Idoso (CRASI) da macrorregião norte do estado de Minas Gerais, Brasil. O grupo avaliado é relativamente homogêneo quanto às características socioeconômicas e demográficas; porém, foram registradas diferenças estatisticamente significativas entre as pessoas idosas do sexo masculino e feminino em relação ao estado conjugal, número de filhos e arranjo domiciliar.

O perfil de saúde dos idosos revela um importante percentual de dependência em capacidade funcional e autonomia, considerando o comprometimento das atividades básicas e instrumentais de vida diária. O relato de multimorbidades e o uso frequente de cinco ou mais medicações (polifarmácia) também são condições que alertam para a necessidade de cuidados especiais em relação ao idoso pós-COVID-19, em função da vulnerabilidade clínica inerente^(3, 4)^.

Pela característica do estudo, não foi possível verificar se as morbidades referidas iniciaram-se antes ou após o quadro confirmado da infecção pelo SARS-CoV-2. Ainda assim, vale salientar que uma metanálise conduzida a partir da avaliação de estudos longitudinais constatou que a COVID-19 pode precipitar o surgimento de algumas condições mórbidas^(6)^.

Constatou-se um maior percentual de mulheres sem companheiros, com maior número de filhos e que residem sozinhas, quando comparado aos idosos do sexo masculino. De modo similar, outros estudos que abordam o processo de senescência também registraram que mulheres idosas sem companheiros são mais comuns do que homens^(24, 25)^.

Em relação às condições de saúde, os resultados encontrados também mostram distinções entre os sexos no que tange à funcionalidade, observando-se uma maior proporção de mulheres com algum comprometimento funcional, o que também é registrado em outras investigações^(24, 26)^.

Adicionalmente, a polifarmácia, outro fator relevante correlacionado à saúde dos idosos, também mostrou-se associada ao sexo feminino, com maiores índices de uso de cinco ou mais medicamentos pelas mulheres. Verifica-se uma consonância com a literatura, pois um estudo longitudinal realizado em 70 municípios brasileiros constatou significativas disparidades entre homens e mulheres idosas, com proporções consideravelmente maiores de polifarmácia entre as mulheres^(27)^.

Outros estudos desenvolvidos na Europa, nos Estados Unidos e no Brasil alertam que a polifarmácia é uma condição comum entre os idosos e, muitas vezes, ocorre de forma associada a múltiplas condições crônicas. Além disso, observa-se uma significativa prevalência de desfechos negativos em decorrência desta condição, como maiores registros de mortalidade, quedas, hospitalizações, reações medicamentosas e aumento dos custos com a saúde^(28, 29, 30)^.

O modelo multivariado revelou que o comprometimento cognitivo, a presença de cinco ou mais morbidades e o comprometimento das AIVDs apresentaram-se como variáveis estatisticamente associadas ao estado de fragilidade. A associação encontrada entre fragilidade e comprometimento cognitivo é consistentemente registrada na literatura, a qual alerta sobre a necessidade da detecção precoce de alterações cognitivas para a compreensão de sua evolução dinâmica e suas repercussões para o estado clínico-funcional do idoso^(31, 32, 33)^.

Nessa direção, um estudo de coorte prospectivo conduzido com idosos em um centro acadêmico de medicina em São Paulo observou uma maior proporção de idosos fragilizados entre aqueles com alteração cognitiva, sendo essa parcela superior à observada entre idosos sem essa condição. Além disso, os autores alertam que pacientes frágeis e com cognição prejudicada apresentam maior risco de mortalidade (*Hazard Ratio* - HR: 4,38; IC95%: 1,95-9,87)^(33)^.

O declínio da capacidade cognitiva é frequentemente verificado com o avanço do processo de envelhecimento, podendo ser impulsionado por fatores genéticos, socioeconômicos, hábitos de vida e a presença de comorbidades. Nesse contexto, a fragilidade tem sido apontada, internacionalmente, como uma condição relevante. Um estudo de coorte longitudinal realizado na Ásia registrou que pessoas idosas com declínio cognitivo demonstraram uma probabilidade 3,77 vezes maior (*Odds Ratio*) de desenvolver fragilidade clínico-funcional (IC95%: 1,42-9,99)^(34)^.

Destaca-se que ambas as alterações compartilham os mesmos mecanismos fisiopatológicos de ativação inflamatória e desregulação neuroendócrina, o que, consequentemente, contribui para que as duas condições coexistam^(34)^. Assim, idosos que apresentam comprometimento cognitivo são considerados em risco para fragilização^(31, 32, 33, 34, 35)^.

Semelhante a este estudo, uma investigação transversal conduzida com pacientes idosos atendidos em um centro especializado em gerontologia no interior de São Paulo constatou uma correlação entre fragilidade e maior número de morbidades (r=0,539; p=0,004)^(31)^. A presença de multimorbidades está ligada a uma maior restrição de atividades de vida diária. Embora não signifique necessariamente a fragilização de um indivíduo, com o avançar da idade, esse fator pode acelerar o desenvolvimento da fragilidade e indicar maior risco para a instalação dessa síndrome^(36)^.

Outra variável que também demonstrou associação estatisticamente significativa com a fragilidade foi o comprometimento das AIVDs. Um estudo transversal conduzido com idosos em São Paulo reforçou essa associação ao concluir que idosos mais fragilizados apresentam piores condições de funcionalidade em relação aos idosos menos fragilizados (r=-0,57; p=0,002)^(37)^. Esses achados indicam que, de modo geral, à medida que a transição para o estado de fragilidade avança, as pessoas idosas exibem pior desempenho nas atividades básicas e instrumentais de vida diária^(38)^.

Ressalta-se que uma parcela significativa dos idosos investigados no presente estudo está inserida no estrato pré-frágil. Considera-se que esses idosos estão em processo de fragilização, o que é um fator preocupante, uma vez que não há uma definição clara sobre o caráter evolutivo da condição. É imprescindível que os profissionais de saúde promovam maior vigilância e cuidado integrado a esse grupo, pois um estudo de coorte longitudinal, desenvolvido em um centro ambulatorial de especialidade geriátrica em Minas Gerais, acompanhou pacientes idosos durante doze meses e constatou a transição para a fragilidade em 33,3% das pessoas idosas não frágeis com comprometimento cognitivo^(32)^.

Analisando de forma particular o contexto da COVID-19, vale ressaltar que a maioria do público idoso neste estudo apresentou situação vacinal incompleta e uma parte considerável do grupo avaliado necessitou de hospitalização em decorrência da doença. Não foi possível avaliar o impacto da doença de forma direta sobre o estado de fragilidade, considerando que os pacientes não foram avaliados previamente.

Em princípio, seria esperado uma maior proporção de idosos frágeis envolvidos no estudo, considerando que outras produções apontam uma relação estreita entre fragilidade e COVID-19^(6, 7, 15, 16)^. No entanto, é importante ponderar que idosos mais frágeis apresentaram desfechos graves, com hospitalizações prolongadas e óbito pós-COVID-19^(3, 7, 16)^. O processo de alocação dos idosos envolvidos neste estudo é um fator limitante para considerações mais específicas sobre a relação entre COVID-19 e fragilidade, pois foi realizado a partir de uma demanda específica e voluntária.

No contexto da pandemia de COVID-19, investigações internacionais alertam que a condição de fragilidade clínico-funcional apresentou correlação com maior risco de desenvolver formas graves da doença, sequelas pós-infecção e/ou com agudização e descompensação de doenças crônicas entre pacientes idosos, particularmente quando atrelada à hospitalização^(1, 3, 7)^. Outros inquéritos destacam ainda que a internação hospitalar de pessoas idosas frágeis afetadas pela COVID-19, com multimorbidades, declínio cognitivo e comprometimento da funcionalidade, repercute em diminuição significativa da sobrevivência^(3, 7, 33, 39, 40).^

### Limitações do estudo

Os resultados do presente estudo devem ser considerados à luz de algumas limitações. Como já mencionado, trata-se de uma série de casos, com análises pontuais, que não permitem a determinação de causalidade. Além disso, deve-se considerar os vieses de memória e de seleção, tendo em vista que os dados da pesquisa foram obtidos por meio do sistema de registros de atendimentos do serviço, os quais foram registrados a partir do relato dos idosos assistidos no centro especializado para avaliação clínica após a infecção por COVID-19. Como as avaliações ocorreram após a instalação da doença, referem-se apenas às pessoas idosas sobreviventes e que estavam aptas a comparecer ao serviço para atendimento, o que compromete uma generalização sobre a condição estudada.

### Contribuições para a área da enfermagem, saúde ou política pública

Apesar das limitações, os resultados observados reforçam a urgência de um cuidado integral ao público idoso, direcionado às particularidades da síndrome da fragilidade e à condição pós-COVID-19. Além disso, reiteram a necessidade de educação permanente e apoio técnico-pedagógico (matriciamento) aos serviços e profissionais de saúde envolvidos nesse contexto de assistência.

## CONCLUSÕES

O perfil de saúde do grupo é caracterizado, majoritariamente, por idosos que apresentam limitações em funcionalidade e autonomia, além de um elevado percentual de morbidades associadas. Predominantemente, o grupo avaliado encontrava-se em condição frágil/pré-frágil.

As variáveis que se mostraram associadas à fragilidade em idosos pós-infecção pelo SARS-CoV-2 foram: comprometimento cognitivo, presença de cinco ou mais morbidades e comprometimento das atividades instrumentais de vida diária.

Portanto, torna-se relevante avaliar a condição pós-COVID-19 como um potencial marcador adicional de fragilização, especialmente entre os idosos. Esses resultados ressaltam a necessidade de uma coordenação do cuidado integrado bem estabelecida e preparada para atender as demandas da população idosa pós-COVID-19.
